# A high throughput approach for analysis of cell nuclear deformability at single cell level

**DOI:** 10.1038/srep36917

**Published:** 2016-11-14

**Authors:** Menekse Ermis, Derya Akkaynak, Pu Chen, Utkan Demirci, Vasif Hasirci

**Affiliations:** 1 BIOMATEN, METU Centre of Excellence in Biomaterials and Tissue Engineering, 06800, Ankara, Turkey; 2 METU Department of Biomedical Engineering, 06800, Ankara, Turkey; 3 Department of Mechanical Engineering, Massachusetts Institute of Technology, 77 Massachusetts Avenue, Cambridge, MA 02139, USA; 4 Bio-Acoustic-MEMS in Medicine (BAMM) Laboratory, Canary Center at Stanford for Early Cancer Detection, Department of Radiology, School of Medicine, Stanford University, Palo Alto, CA, 94304, USA; 5 Department of Electrical Engineering (by courtesy), Stanford University, Stanford, CA, USA; 6 METU Department of Biological Sciences, 06800, Ankara, Turkey

## Abstract

Various physiological and pathological processes, such as cell differentiation, migration, attachment, and metastasis are highly dependent on nuclear elasticity. Nuclear morphology directly reflects the elasticity of the nucleus. We propose that quantification of changes in nuclear morphology on surfaces with defined topography will enable us to assess nuclear elasticity and deformability. Here, we used soft lithography techniques to produce 3 dimensional (3-D) cell culture substrates decorated with micron sized pillar structures of variable aspect ratios and dimensions to induce changes in cellular and nuclear morphology. We developed a high content image analysis algorithm to quantify changes in nuclear morphology at the single-cell level in response to physical cues from the 3-D culture substrate. We present that nuclear stiffness can be used as a physical parameter to evaluate cancer cells based on their lineage and in comparison to non-cancerous cells originating from the same tissue type. This methodology can be exploited for systematic study of mechanical characteristics of large cell populations complementing conventional tools such as atomic force microscopy and nanoindentation.

One of the elementary questions of cell fate and morphogenesis is how critical the role of nuclear shape is in these processes. Nuclear shape is preserved by nuclear lamins and the cytoskeletal elements^1^,^2^. Mechanical properties of both the nucleus and the cell also contribute to the nuclear shape and elasticity^3^. The state of health of the cell influences this interrelation. A tool for quantifying nuclear deformability would help study the intrinsic differences between various cell categories and also heterogeneity in a cell population.

It is suggested that reduction or absence of expression of lamin A/C; type-V intermediate filaments of nuclear lamina^2^; is a common feature in a variety of cancers including small cell lung cancer (SCLC), skin basal cell and squamous cell carcinoma, testicular germ cell tumor, prostatic carcinoma, leukemia, and lymphomas^4^,^5^,^6^,^7^,^8^. The reduction in lamin A/C expression is associated with cancer subtypes, aggressiveness, proliferative capacity and differentiation state^8^. In the case of depletion of components of the Linker of Nucleoskeleton and Cytoskeleton (LINC) complex; which connects the nuclear lamina and the nuclear membrane to the cytoskeleton^3^; such as nesprins and SUN proteins, aberrations of nuclear shape and softening of the nucleus and the cytoplasm were observed^9^. The nuclear lamina and LINC complex molecules have crucial roles in collective 2D migration and maybe metastasis. When mechanical properties of healthy and cancer cells were compared in biophysical settings it was consistently shown that cancer cells were found to be softer and this was related to increased metastatic potential^10^. All this information points to the importance of nuclear deformability in cancer and might contribute significantly to our understanding of cancer.

Micro- and nanoscale engineering technologies present unique opportunities to study the effects of substrate surface cues on cellular processes like differentiation, carcinogenesis, epithelial to mesenchymal transition or metastasis. For instance, our earlier studies^11^,^12^,^13^,^14^ and those from others^15^,^16^,^17^,^18^,^19^,^20^,^21^,^22^,^23^,^24^,^25^ used random or controlled distribution of topological surface features, such as pits, protrusions^19^,^20^ channels or pillars^16^,^17^,^18^,^26^,^27^, to induce changes in alignment, and deformation of cellular and nuclear shape. Follow up studies showed that the extent of changes in the nuclear morphology of adherent cancer and other cell types differ when grown on substrates decorated with nano and microstructures^13^,^14^,^24^,^25^. This phenomenon may be explained in part by the relative mechanical softness of cancer cells^28^,^29^,^30^, a property, which may contribute to their metastatic potential^10^,^31^,^32^,^33^. Systematic analysis of cell nuclei morphology in response to intracellular and extracellular cues may thus provide important insights into differentiation^34^,^35^,^36^, migration^37^,^38^ and attachment of cells^39^, and cancer metastasis^40^,^41^. However, how different cell types respond to topological cues are still not fully discovered^42^,^43^. In addition, it is not clear yet how cells of a single population respond differently to physical and chemical stimuli from the environment, such as those from the topography^44^,^45^. In order to address the causes of heterogeneous cell responses, a method is needed to quantify the level and extent of morphological deformations. Cellular heterogeneity of homogeneous populations is increasingly recognized as a ubiquitous phenomenon^46^,^47^,^48^,^49^. A number of attempts have been made to assess the level of deformation of elastic biological tissues. Although characterization of cytoskeleton deformation has been demonstrated using several techniques like optical stretcher^50^, the quantitative measurement of cell nuclear deformation has not been fully studied especially in a high throughput format. Current methods used in the study of deformability of cell nuclei include optical tweezers, micropipette aspiration, AFM-nanoindentation, microfluidics devices^28^,^29^,^30^,^31^,^32^,^51^,^52^,^53^,^54^. (Sup. Table 1) Recent studies in the literature reported that osteosarcoma cells with different metastatic potentials (MG-63 and Saos-2 cell lines) showed nuclear deformations on physically patterned polymeric surfaces^15^,^16^. The cytoplasms of non-cancerous, immortalized cells, also showed a time dependent deformation and orientation but no nuclear deformation even after 48 h of contact with such surfaces^16^. In a microfluidic system fibroblasts migrated through channels with of 2, 3 and 5 μm constrictions and results showed that Lamin A/C deficient cells had higher nuclear deformability and were better at migrating through narrow constrictions^37^. A similar phenomenon in 3D porous matrices was observed and it was shown that migration depends on pore size of the ECM and nuclear deformability of cells^55^. Human embryonic stem cells in terminal differentiation were found to become less deformable against micromanipulation and stiffen up to 6 times^56^. In a micropipette aspiration study, melanoma cell invasion slowed down after stiffening of the nuclei due to overexpression of a form of Lamin A that is found in diseased and aged cells^54^. The epithelial monolayers were exposed to mechanical strain and showed that actin and microtubules play a key role in nuclear deformations^57^. All these studies showed that deformability of cell nucleus has an impact on cellular processes, metabolism and response of cells to their microenvironment. However, a thorough study of a method to quantify deformability of cell nucleus at single cell level and for population-wise analysis in a high-throughput manner is still needed.

Here, we present the Micropillar Induced Nuclear Deformation (MIND) platform consisting of microengineered cell culture substrates and image analysis algorithms (Fig. 1a). Micropatterned cell culture substrates induce changes in cellular and nuclear morphology. Our custom made, high content image analysis algorithm profiles the cell populations and changes in the morphology of individual nuclei at the single cell level. This approach has the potential to conduct systematic studies of cell spreading, deformation, differentiation, biochemical and genetic profiling of heterogeneous cell populations.

## Experimental Methods

### Fabrication of SU-8 micropillar array chip

We fabricated SU-8 micro-pillar array chips using a standard photolithography procedure (Sup. Fig. 1). Briefly, a fresh silicon wafer (4-inch, University Wafer, MA) was used as the substrate. The silicon wafer was first cleaned in organic solvents (acetone for 15 s, isopropanol for 15 s), dehydrated (185 °C, 5 min), cooled down with nitrogen, and further cleaned by oxygen plasma (oxygen flow rate: 20 cm^3^.s^−1^; chamber pressure: 380 mTorr; power: 150 W, 3 min). Subsequently, the wafer was spin-coated with OmniCoat™ (MicroChem) (13 nm, 3000 rpm, 30 s) and cured (200 °C, 1 min) to improve SU-8 adhesion to the substrate. Next, the wafer was spin coated with a 10 μm thick layer of SU-8 2100 photoresist (MicroChem) (1900 rpm, 45 s with a ramp rate of 500 rpm.s^−1^), baked (65 °C for 1 min, and 95 °C for 2 min) and performed with edge bead removal. The SU-8 was exposed to UV (i-line, 140 mJ.cm^−2^ using a SUSS MA6 Mask Aligner) through a custom designed photomask (Fineline Imaging, CO). Photomask was tightly pressed on the solidified photoresist layer during the UV exposure to achieve an undistorted pattern transfer. The UV-exposed SU-8 was baked on a hot plate (65 °C for 1 min and 95 °C for 3 min) and gently washed with SU-8 developer (MicroChem) to remove uncrosslinked photoresist. SU-8 development time and hydrodynamic shear stress were carefully controlled to avoid destroying micropillars with high aspect ratio. Finally, the developed SU-8 structure was hardened by baking (175 °C, 5 min) and slowly cooled down to room temperature. The fabricated SU-8 structure was coated with a 10 μm layer of positive photoresist (S1822, Shipley Microposit) as a protective layer before dicing. The SU-8 patterned wafer was cut into 12 mm × 12 mm chiplets with an automatic dicing saw (Model DAD 321, DISCO, Japan) with a custom defined program. After cutting, the protective layer on the SU-8 structure was removed by successively washing with acetone and isopropanol, and the chiplets were dried with nitrogen for final use. Nine 3-D micropatterned chiplets decorated with a combination of square prism pillars with different sizes (4 × 4 μm^2^ (P4), 8 × 8 μm^2^ (P8), and 16 × 16 μm^2^ (P16)) and interpillar distances (4 μm (G4), 8 μm (G8) and 16 μm (G16)) were manufactured. Thus, the array consists of the following surfaces: P4G4, P4G8, P4G16, P8G4, P8G8, P8G16, P16G4, P16G8 and P16G16. A control chiplet with no pillars was used as an unpatterned control (Control).

### Preparation of poly(lactic acid-*co*-glycolic acid) (PLGA) (85:15) micropatterned films

Negative copies of the wafers were moulded using polydimethylsiloxane (PDMS), prepared from Sylgard 184 silicone polymer and Sylgard 184 Curing agent (Dow Corning Company, UK) mixed in a ratio of 10:1 (w/w). The silicone pre-polymer mix was poured onto the patterned surface of the wafer in a petri plate; vacuum was applied for 45 min and then heated (70 °C, 4 h). After cooling, the formed PDMS structure was peeled off from the wafer producing a negative copy of the original (Sup. Fig. 2). This negative mould was used to make poly(lactic acid-*co*-glycolic acid) (PLGA) films. A PLGA 85:15 (For You Company, China) solution in chloroform (6%, w/v) was prepared, poured onto the patterned PDMS template and air-dried for 36 h (Sup. Fig. 2). To prepare smooth PLGA surfaces, unpatterned PDMS moulds were used. The films were stored on Teflon sheets in a desiccator at room temperature before use. PLGA substrates were used without any coating.

### Characterization of the PLGA films using SEM

Patterned surfaces of the films were coated with Au–Pd under vacuum and examined with an SEM (400 F Field Emission SEM, USA).

### *In vitro* experiments

The Ethical Committees of both Gulhane Military Medical Academy and the Middle East Technical University approved all experimental protocols. The methods were carried out in accordance with the approved guidelines. All experiments were conducted after obtaining written informed consent from all the subjects.

Saos-2 cells (Passages 17 and 25, No: HTB-85, ATCC) were cultured at 37 °C in a humidified atmosphere with 5% CO_2_ in RPMI 1640 (Lonza, USA) supplemented with 10% foetal bovine serum (FBS) (Lonza, USA), 100 U.mL^−1^ penicillin (Sigma, USA), and 100 μg.mL^−1^ streptomycin (Sigma, USA). L929 cells (Passages 10 and 15, No: CCL-1, ATCC) were cultured at 37 °C in a humidified atmosphere with 5% CO_2_ in DMEM (Lonza, USA) supplemented with 10% FBS, 100 U.mL^−1^ penicillin, and 100 μg.mL^-1^ streptomycin. SH-SY5Y cells (Passages 15 and 25, No: CRL-2266, ATCC) and MCF7 cells (Passages 15 and 20, No: HTB-22, ATCC) were cultured at 37 °C in a humidified atmosphere with 5% CO_2_ in DMEM Low glucose (Lonza, USA) supplemented with 10% FBS, 100 U.mL^−1^ penicillin, and 100 μg.mL^−1^ streptomycin. Isolation of human osteoblast-like cells (hOB) were performed using bone fragments harvested from elective joint replacement surgery patients. Fresh bone specimens were then transferred to sterile growth media and transported to the cell culture laboratory. Bone fragments were washed with phosphate buffered saline (PBS) and serum-free DMEM growth medium and cut into 2–3 mm pieces. Pieces were transferred to T175 flasks and supplemented with growth medium. They were cultured without disturbing for 7 days and afterwards the growth medium was replaced every 4 days. Migration of cells from bone matrix to TCPS surface was studied with an inverted phase contrast microscope. Cells were further subcultured in McCoy-5A (Lonza, USA) medium supplemented with ascorbic acid and L-glutamine (Lonza, USA). When 90% confluence was reached the cells were trypsinised and either passaged further or cryopreserved until use. Micropatterned PLGA films were sterilized by exposing both sides to UV in a laminar flow hood for 25 min and then placed in a 12 well tissue culture plate. Cells suspended in growth media were seeded at a density of 5,000 cells/film. After 1 h incubation for cell adhesion, 2 mL of growth medium was added into each well and plates were incubated at 37 °C and 5% CO_2_.

### Microscopy

Micropatterned films of PLGA were removed from the growth medium and washed twice with PBS; cells were fixed in 4% paraformaldehyde and permeabilised with 1% Triton-X 100 solution (Applichem, Germany). Specimens were incubated in PBS containing 1% Bovine Serum Albumin (BSA) (Sigma-Aldrich, USA) at 37 °C, for 30 min in a humidified 5% CO_2_ incubator to block nonspecific binding. After blocking, specimens were incubated for 1 h at 37 °C with Alexa Fluor 532 Phalloidin (Invitrogen, USA) to stain the actin in the cytoskeleton, and 5 min at room temperature with DAPI (Invitrogen, USA) or DRAQ5 (Abcam, UK) to stain the nuclei. Fluorescence micrographs of the cells were obtained using an upright fluorescence microscope under 350 nm, 488 nm, 550 nm or 630 nm LED sources and appropriate filter sets (Zeiss Axio Imager M2, Germany) or with an upright confocal laser scanning microscope (CLSM) under 488 nm, 532 nm, 630 nm lasers (Leica DM2500, Germany). SEM specimens were washed twice with PIPES (piperazine-N,N′-bis(ethanesulphonic acid)) buffer (Sigma Aldrich, USA), and fixed in 4% paraformaldehyde solution for 5 min. After washing with PIPES buffer, the samples were stained with 1% osmium tetroxide (OsO_4_) (Sigma Aldrich, USA), washed twice with PIPES buffer and dehydrated by immersing in an ethanol series. Cell seeded films were coated with Au–Pd under vacuum and examined with the SEM (400 F Field Emission SEM, USA).

### Image-based quantification of cell deformation

**Figure 1:**
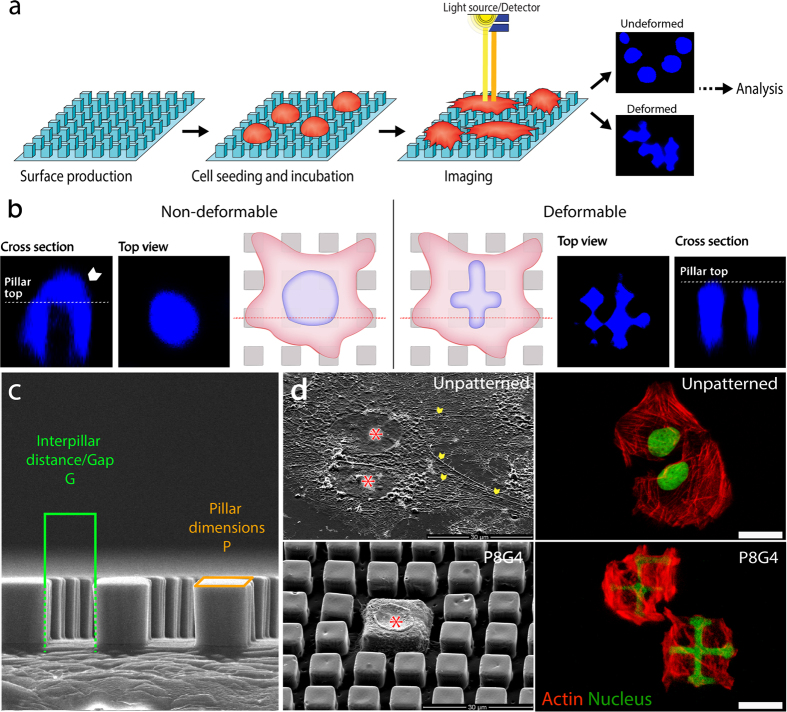
Micropillar induced nuclear deformations (MIND) approach to cancer cell identification. (**a**) Micropatterned surfaces were produced using photolithography, and then solvent casting. Cells were seeded and incubated, followed by fixation and fluorescent tagging of DNA, and imaged by a fluorescence microscope. Micrographs were analysed using a custom made algorithm. (**b**) When cultured on micropatterned surfaces, non-deformable (b1) and deformable (b2) cells responded to surface topography differently. Nucleus of a non-deformable cell when observed from above, was round or oval (*top view*) and from side (*cross section*) a thick portion of the nuclear material sat on top of the pillar. Nucleus of a deformable cell completely flowed into the gaps between the pillars, undergoing extensive deformations (*cross section*) and no nuclear compartment could be observed from the top of a pillar (*top view*). (**c**) We manufactured a micropillar array with 9 fields. Each field was named based on the dimensions of the pillars (P) and interpillar spacings/gaps (G) it was decorated with. For example, a surface with 8 × 8 μm^2^ pillars and 4 μm gaps was named P8G4. (**d**) On the SEM micrographs of the Saos-2 human osteosarcoma cells, asterix show the nuclei and chevrons show the stress fibres formed. The cell body on the micropatterned substrate was located on top of 4 adjacent pillars while the nuclear envelope is seen in the centre. Confocal images of Saos-2 cells on smooth and on P8G4 micropatterned surface are also presented to support the SEM observations (Stains: Red: Actin cytoskeleton/Alexa532 Phalloidin; Green: Nucleus/DRAQ-5) (scale bar 25 μm). Cell nuclei filling the gaps between the 4 pillars were observed showing that most of the nuclear material conforms to the shape of the gap.

Fluorescence micrographs were obtained using a Zeiss Axiovert M2 microscope equipped with x63 water immersion objective and analysed using a custom program written using MATLAB (Mathworks Inc., Natick MA, USA) to quantify the extent of nucleus deformation on each patterned surface (Fig. 1a, Sup. Fig. 4). Original images were in Red (R), Green (G), Blue (B) format, and had width (W) and height (H) of 2452 and 2056 pixels, respectively. For faster processing, images were resized to 30% of their original dimensions. Only the B channel, which contained the most information, was kept to obtain a grey scale image (Ig).

### Image pre-processing

We performed a number of image quality checks. First, cells with standard deviation of grey scale intensities greater than 0.5 were eliminated. These were generally out of focus cells or were unevenly illuminated due to vignetting. Then, images were binarized (Ib) using Otsu’s method^58^. Cells that were attached to any of the four boundaries of the image were also eliminated, because only a fraction of these cells were visible in the photographs, and it was not possible to assess their true shape or deformation. Following this, we categorised cells based on size and those with areas that were too small (fewer than 50 pixels) or too big (larger than 1% of image H × W) were removed. As the final step, cells that were going through mitosis were identified using watershed transform^59^ and excluded from analysis. The remaining cells were smoothed using a Gaussian filter (n = 5, σ = 10) to even pixel roughness on the edges, and morphological opening was performed to eliminate any artefacts. To achieve rotation invariance, each arbitrarily oriented cell was rotated to a common orientation^60^ by aligning its major axis with the y-axis, and ensuring the centroid was always on the right side of the major axis. For scale invariance, each cell was resized so that its largest dimension (H) was 64 pixels long, and its width was scaled accordingly to maintain the original aspect ratio. As a final data quality check, we re-filtered the cells with the same Gaussian kernel, followed by a median filter (n = 3) to eliminate salt-and-pepper noise, and also excluded cells whose pixels occupied 100% of their bounding boxes; these were likely to cause residual image processing artefacts.

### Feature extraction

After pre-processing the images, we extracted two scale, rotation and translation invariant features from binary images of cells: rectangularity and circle variance. Rectangularity (R) is the ratio of the area of a cell to that of the minimum rectangle that encompasses it, and is a measure of the compactness of a cell. The rectangularity of an ideal circle is 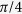, and we subtracted this value from the rectangularity of all cells so an ideal circle would have a value of zero:


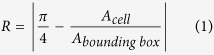


Circle variance (CV) is the ratio of the standard deviation (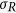) of the radial Euclidean distance (d_i_) between the centroid of a cell to each of its N boundary points, to that of their mean (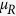), where:


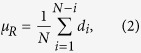


and


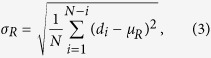


yielding


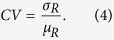


Here, the Euclidean distance d_i_ in the radial direction is defined as:


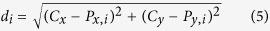


where (*C*_*x*_, *C*_*y*_) represents the center of the cell, and (*P*_*x*_, *P*_*y*_)_*i*_ is the i^th^ point on its boundary.

Circle variance is zero for an ideal unit circle as all distances *d*_*i*_ are equal to the radius of the circle, with their standard deviation being 0, and mean 1. In addition to being scale, rotation and translation invariant, use of these two simple descriptors has the advantage that data can easily be visualized using two-dimensions without the need for dimensionality reduction. Further, they have low computational complexity, allowing for inexpensive hardware implementations.

### Surface selection

**Figure 2:**
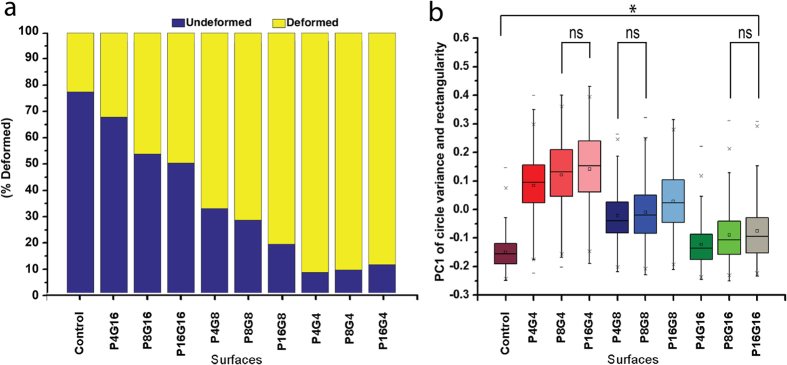
Deformation frequency and PC1 of Saos-2 cells on 10 surfaces tested. Two descriptors, circle variance and rectangularity were used to characterize the extent of deformation on the surfaces. (**a**) Based on the test data, five regions were identified in the rectangularity-circle variance space that quantified the degree of nuclear deformation. Among these, regions 1 and 2 (cut off: circle variance, rectangularity ≤0.2) represented minimal deformation. After constructing circle variance versus rectangularity plot, the region with the most circular nuclei was labelled as Undeformed and rest as Deformed region. For each surface, fraction of the nuclei falling in Undeformed and Deformed regions were calculated. Control surface had the highest (77.5%) Undeformed nuclei fraction while P4G4 had the lowest fraction (8.60%). (**b**) Principal component analysis was conducted for circle variance and rectangularity descriptor measurements and Principal Component 1 (PC1) results were further analysed statistically (*p < 0.05, ns: non-significant). Distributions of cell nuclei PC1 were not normal according to Kolmogorov-Smirnov test so Welch’s ANOVA with Games-Howell Test was used (p < 0.05) to show significance. All nine patterned surface shape distributions were statistically significant compared to the Control surface. Also distributions of surfaces with different gap sizes were found to be significant. In the 4 μm gap group (P4G4, P8G4, and P16G4) only the distribution of P4G4 was significantly different than the other two, and among all the surfaces, P4G4 had the highest significant difference with the Control (*μ*_*x* control_ = −0.150648, *μ*_*x* P4G4_ = 0.084128).

For the purpose of identifying the surface type that induced maximal nucleus deformation, we used micrographs of Saos-2 cells, which were known to undergo extreme deformations^61^, seeded on Control, P4G4, P4G8, P4G16, P8G4, P8G8, P8G16, P16G4, P16G8 and P16G16 surfaces. Following the pre-processing steps as described (Image pre-processing), we extracted CV and R from each cell and categorised them as undeformable if R ≤ 0.2 and CV ≤ 0.2, and deformable if R > 0.2 and CV > 0.2 (Fig. 2). Kolmogorov-Smirnov test was used to check the normality of distributions. Samples did not show normal distribution and revealed heteroscedastic distribution. Analyses were conducted using non-parametric Welch’s ANOVA test for the first principal component of R and CV. For pairwise comparisons of samples Games-Howell *post hoc* test was used (p < 0.05).

### Nuclear deformation on the P4G4 surface

**Figure 3:**
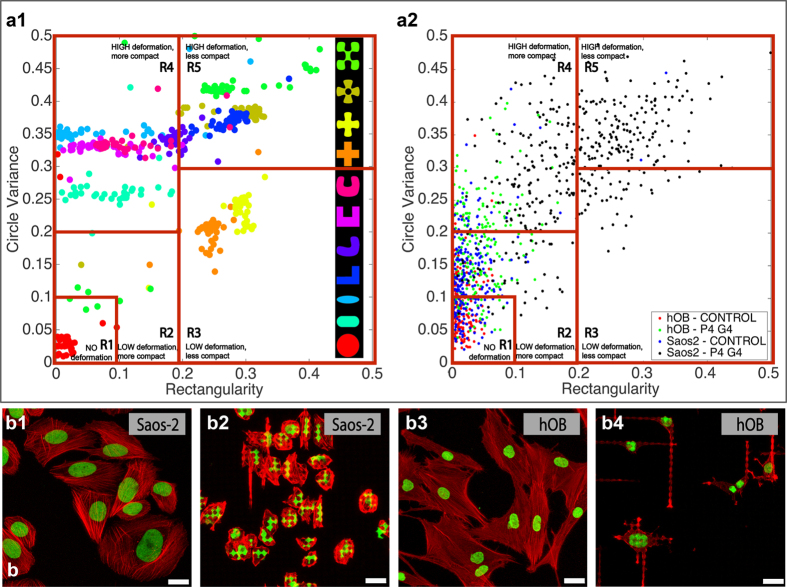
Test images were used to identify five regions of deformation based on rectangularity (R) and circle variance (CV), which were then applied to Saos-2 and hOB data. CLSM images show nuclear morphology of Saos-2 and hOB cells tested. (**a**) R and CV values were calculated from 11 artificially generated templates mimicking nuclear deformations of actual cancer cells (*a1*). 50 images of each template with various orientations, scale, and software-generated noise were tested. This artificially generated population was processed by our software and the resulting distribution of R and CV values were used to identify 5 main regions of deformation: R1 (R_1_, CV_1_ ≤ 0.1, 0.1): no deformation, R2 (0.1, 0.1 < R_2_, CV_2_ ≤ 0.2, 0.2): low deformation-more compact, R3 (0.2, 0 < R_3_, CV3 ≤ 0.5, 0.3) low deformation-less compact, R4 (0, 0.3 < R_4_, CV4 ≤ 0.2, 0.5) high deformation-more compact and R5 (0.2, 0.3 < R_5_, CV_5_ ≤ 0.5, 0.5) high deformation-less compact. (*a2*) Using the regions identified in (*a1)*, the average nuclear deformability of a population was quantified through a *deformation score (DS)* calculated by assigning each cell a weight based on the region it fell in (R1 = 1, R2 = 2, R3 = 3, R4 = 4, R5 = 5), and then these weights were averaged for all cells in each population. A deformation score less than or equal to 3 indicates little or no deformation and that the population is non-cancerous. The R and CV values for Saos-2 and hOB cells cultured on Control and P4G4 surfaces are: for hOB, DS = 1.04 on Control and 1.22 on P4G4, and for Saos-2, DS = 1.58 on Control and 3.55 on P4 G4. On the P4G4 surface, 97% of individual hOB cells had DS ≤ 3, and 70% of individual Saos-2 cells had DS > 3. On the Control surface, 99% of hOB cells and 96% of Saos-2 cells had DS ≤ 3, meaning that they were minimally deformed or not deformed at all. (**b**) Confocal micrographs of Saos-2 nuclei on Control (*b1*) show no deformations while on P4G4 surface (*b2*) they show extensive deformations resulting in lobulated “t” and “+” like shapes. Micrographs of hOB nuclei reveal that they do not deform on Control (*b3*) and minimally deform on P4G4 (*b4*) surfaces.

Using the P4G4 surface, which induced the highest level of nuclear deformation, we evaluated the diagnostic performance of our software algorithm using five different cell types: L929, Saos-2, hOB, MCF-7, and SH-SY5Y. To accurately distinguish ‘*Non-Deformed*’ cell populations from ‘*Deformed*’ ones, we developed a finer scoring rubric than the one we used for surface selection. We defined a *deformation score (DS)*, which combined how much the shape of a cell deviated from an ideal circle with whether it stayed more compact (e.g., like an ellipse) or obtained a bent shape (e.g., the shape of the letter “L”). To derive the parameters of this rubric, we created a database of synthesized cell nuclei. We designed 11 main cell *templates* (i.e., variants of deformed cells) we expected to see on cells on the P4G4 surface, with 50 examples of each (550 cells total) (Fig. 3a1). Each cell had a random orientation and scale, and was randomly filtered using Pinch, Twirl, Wave and Ripple filters in Photoshop (Adobe, Inc.) to distort and add noise, simulating actual data. Then, based on the distribution of these test data in the two-dimensional rectangularity and circle variance space, we performed gating to obtain the five regions. The regions were characterized as follows:

R1 (R_1_, CV1 ≤ 0.1, 0.1): no deformation,

R2 (0.1, 0.1 < R_2_, CV2 ≤ 0.2, 0.2): low deformation-more compact,

R3 (0.2, 0 < R_3_, CV3 ≤ 0.5, 0.3) low deformation-less compact,

R4 (0, 0.3 < R_4_, CV4 ≤ 0.2, 0.5) high deformation-more compact and

R5 (0.2, 0.3 < R_5_, CV_5_ ≤ 0.5, 0.5) high deformation-less compact (Fig. 3a1).

After defining the regions as above, we assigned weights to each cell based on the region it fell in *w*_*i*_ = 1:5, where *i* = *R*1 to *R*5. These weights indicate the deformation score at an individual cell level and are used as input to calculate population-level deformation score as follows:


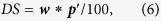


where ***p*****′** is a vector representing the percentage of cells that fall in each of the regions R1–R5. An undeformable cell population that have minimal or no deformation of the nuclei (e.g., theoretically, when all the cells would fall into gating area #1) would receive the minimum score *DS* = 1. Similarly, a deformable cell population that have extensively deformed nuclei (e.g., theoretically, when all the cells would fall into gating area #5) would receive the maximum score *DS* = 5. Based on this convention, the *‘Non-Deformed*’ and ‘*Deformed*’ classes in the Surface Selection section correspond to DS ≤ 3 and DS > 3, respectively. We adopted this threshold as the cut-off between populations with deformable and undeformable nuclei.

We quantified morphological differences in cell nuclei using two features extracted from binary images: rectangularity and circle variance (Equations 1, 2, 3, 4, 5). Rectangularity measures the ratio of the area a cell occupies relative to the area of the smallest bounding rectangle that fits around it, and is a measure of compactness. Circle variance is the ratio of the standard deviation to the mean of the distribution of distances from the centroid of the cell to each point on its perimeter. When combined, these two features quantitatively show how much a cell deviates from an ideal circle (circularity), and how it deviates from an ideal circle (rectangularity): is the morphology compact like an ellipse, occupying most of its bounding box, or has it deformed into a less compact shape like an “L”, with some unoccupied space within its bounding box. The range of deformation of severely deformable cells varies from near-circular or elliptical shapes to “#” shapes in the most extreme cases, when the nuclei fill the entire area available to them. Throughout this range, cells can take shapes resembling the letters “T”, “L”, “U”, and “C”. Rectangularity and circle variance, both of which are scale, rotation and translation invariant, are ideal and sufficient features for quantifying deformations of cells on our micropatterned surfaces^62^,^63^.

## Results

### Micropillar induced nuclear deformation (MIND) approach for cancer cell characterisation

Nine 3-D culture surfaces decorated with square prism pillars with different sizes (4 × 4 μm^2^ (P4), 8 × 8 μm^2^ (P8), and 16 × 16 μm^2^ (P16)) and interpillar distances (4 μm (G4), 8 μm (G8) and 16 μm (G16)), and with a fixed height of ~8 μm were manufactured from poly(lactic acid-*co*-glycolic acid) (PLGA) polymer (Fig. 1c, Sup. Fig. 3a,b). Surfaces were designated according to dimensions of surface decorations as P4G4 for 4 × 4 μm^2^ micropillars spaced at 4 μm distance or P4G8 for 4 × 4 μm^2^ micropillars spaced at 8 μm distance. An unpatterned surface was used as the control (Control). PLGA (lactic acid: glycolic acid 85:15) was chosen as the surface material for its slow degradability (~20% weight loss in 6 weeks for high LA:GA were reported^61^) and biocompatibility.

Five different cell types were used for the experiments; Saos-2 cell line that originated from human osteosarcoma, hOB cells that were isolated from healthy patients undergoing elective orthopaedic surgery, SH-SY5Y cell line originating from human neuroblastoma, MCF-7 cell line originating from human non-invasive breast carcinoma, and L929 mouse fibroblast cell line for internal controls. Initially, human osteosarcoma cell line Saos-2 was used for testing the nine micropatterned surfaces. Extent of Saos-2 nuclei deformations varied on different surfaces (Fig. 1d, Sup. Fig. 3c). On some surfaces, their nuclei were round or elliptical, but on others the more radically deformed nuclei took “C” or “+” forms were observed. On the Control surface Saos-2 cell bodies were polygonal and nuclei were round (Fig. 1d top panel, Sup. Fig. 3c). When Saos-2 cells were cultured on P8G4, cell bodies were restricted to the available surface area on top of the pillars (Fig. 1d bottom panel). In the Control, an unobstructed surface was available for spreading while micropatterned surfaces forced the cells to stay close to the pillars and limited their cytoskeletal extension (Fig. 1d top panel, Sup. Fig. 3c).

We calculated the ratio of the pillar top area to the base area as 0.8 regardless of the type of array. The aspect ratios (pillar height to pillar width) were 2.08 for P4 (P4G4, P4G8, and P4G16), 1.04 for P8 and 0.58 for P16 groups. The differences observed in cell responses within the same aspect ratio groups were mainly related with the interpillar distances.

### Quantification of cell nuclear deformation on the micropillar array surfaces

In order to choose the surface that leads to the highest deformation of cell nuclei (according to the DS scale), Saos-2 cells were seeded and cultured on these 9 patterned surfaces and the Control surface for 48 h. Two shape descriptors, *i.e.*, circle variance (CV) and rectangularity (R) were analysed using the fluorescence micrographs of the cell nuclei (Equations 1, 2, 3, 4, 5). Cell nuclei were classified as deformable or undeformable according to the CV and R values; and fraction of the nuclei falling in these regions (R1–5) were calculated using Equations 1, 2, 3, 4, 5 (Fig. 2a). Control surface had the highest (77.5%) undeformed nuclei fraction while P4G4 had the lowest (8.60%) value (Fig. 2a). In order to identify the parameter that leads to the highest nuclear deformation, we performed Welch’s ANOVA analysis on the population means of each surface (Fig. 2b). We used principal component analysis (PCA), a common dimensionality reduction technique^64^ in order to project circle variance and rectangularity onto a single axis that preserve the highest variance. Using this reduced dimension for analysis, the difference between the nuclear deformations of cells on Control and patterned surfaces were found to be statistically significant. When nuclear deformations of the cells on patterned surfaces were compared, it was found that surfaces with the same interpillar spacing (G4, G8 or G16) yielded similar results. Therefore, interpillar spacing appears to be the major factor influencing the level of nuclear deformation (Fig. 2b). In addition, pillar dimensions serve as a minor determinant of the observed differences in nuclear shape. For example, within the 4 μm spacing group (P4G4, P8G4, and P16G4), the surface with the smallest pillar dimensions (P4G4) was significantly different than the other two (p < 0.05). Similarly, mean of P4G4 surface was statistically significant compared to Control (*μ*_*x control*_ = −0.15, *μ*_*x P4G4*_ = 0.084).

After determining the surface that leads to the highest nuclear deformation (i.e., P4G4; *see* Methods- Surface Selection, Nuclear deformation on P4G4 surface) using Saos-2 human osteosarcoma cell line, we tested the P4G4 surface with a pair of cells originating from bone tissue, Saos-2 and human osteoblast like cells (hOB), to evaluate how deformation is influenced by cell type. We defined a *deformation score (DS)*, which combined how much the shape of a cell deviated from an ideal circle with whether it stayed more compact (e.g., like an ellipse) or obtained a bent shape (e.g., the shape of the letter “L”) using R and CV data from image analysis. To derive the parameters of this rubric, we designed a set of 11 cell nuclei deformations (shapes) (Fig. 3a1) 13,14,24. We calculated the R and CV values for these shapes. We then performed gating using these results in the two-dimensional R and CV space, and obtained 5 regions (R1-R5), and assigned arbitrary weights to each cell, based on the region it fell in (R1-R5) (*see* Methods-Nuclear deformation on the P4G4 surface) (Fig. 3a1) and used these values as an input to calculate population-level deformation score (DS) (Equation 6). After gates of the regions R1-R5 were defined, micrographs of Saos-2 and hOB cells cultured on P4G4 and Control were analysed. It was found that 97% of the hOB cells seeded on P4G4 were classified as ‘*Non-Deformed*’ while 70% of Saos-2 cells were classified as ‘*Deformed*’ (Fig. 3a2).

On the Control surface, most cells were found to be ‘*Non-Deformed*’ (hOB 99%, Saos-2 96%). Deformation scores of hOB and Saos-2 cell populations were calculated as 1.04 and 1.22 on the Control, and 1.58 and 3.55 on the P4G4, respectively. These results show that DS of Saos-2 cultured on the P4G4 substrates were at least twice as high as that of hOB, while DS of these cells on Control were similar. These results show the ability of the P4G4 surface to distinguish between Saos-2 and hOB using DS. These results are also supported by the deformations observed in the CLSM micrographs (Fig. 3b).

### MIND analysis of different cell types for identifying cell heterogeneity and distinguishing cancer cells

**Figure 4:**
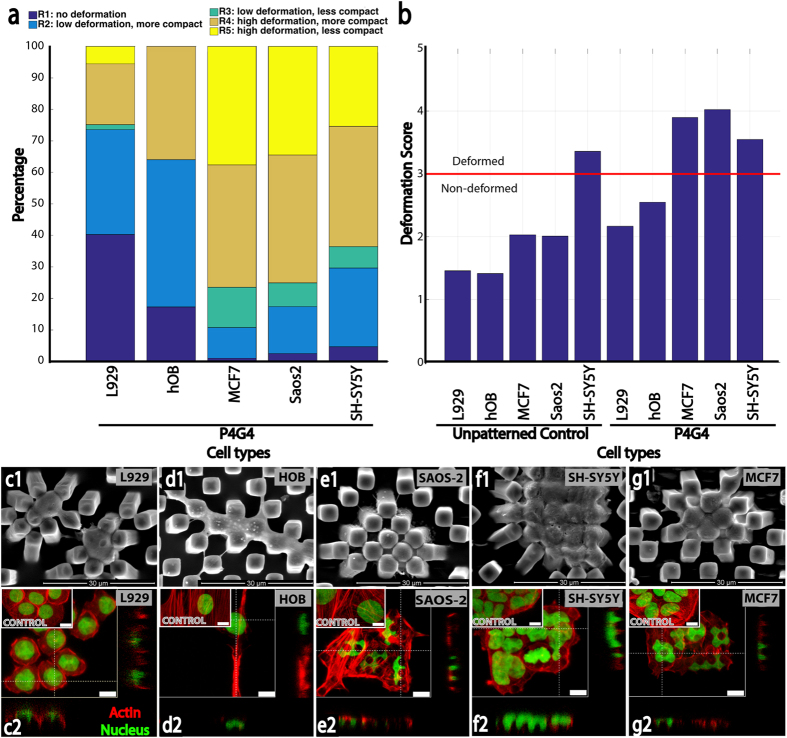
Quantification of deformation of two non-cancerous and three cancer cell types on P4G4 surface. (**a**) Nuclei of the different cell types were categorised according to the regions of CV and R plot. L929 and hOB (non-cancerous cell types tested) had the highest number of nuclei in R1-R2, while SH-SY5Y, MCF7 and Saos-2 (cancerous cell lines) had the highest number of nuclei in R3-R5. Their corresponding deformation scores were L929: 2.17, hOB: 2.54, Saos-2: 4.02, MCF-7: 3.90 and SH-SY5Y: 3.55. (**b**) Comparison of deformation scores of the cells tested on unpatterned control and on P4G4 surfaces. Results show that all cell types except SH-SY5Y had deformation scores lower than 3 on the unpatterned control surface. On the other hand, non-cancerous cell types had deformation scores <3 while cancerous cell lines (MCF7, Saos-2, SH-SY5Y) had scores >3. (**c–g**) P4G4 surface was tested with 5 different cells: fibroblasts (L929), primary bone derived cells (hOB), osteosarcoma cells (Saos-2), neuroblastoma cells (SH-SY5Y), and breast cancer cells (MCF7). SEM images (Scale bars: 30 μm. Stains: OsO_4_) (c1-g1). CLSM images and z-stacks (Scale bars: 10 μm. Stains: Green: Nucleus/, DRAQ5, Red: actin cytoskeleton/Alexa Fluor 532-Phalloidin) (c2-g2).

After comparing the extent of nuclear deformation of Saos-2 with hOB above, types of cells studied was expanded to 5 with the inclusion of 2 other cancer cell lines (MCF-7 human breast adenocarcinoma, and SH-SY5Y human neuroblastoma) and a fibroblast cell line (L929 mouse fibroblasts) (Fig. 4).

Cell nuclei were categorised as was done earlier (*see* Methods- Nuclear deformation on P4G4 surface) into 5 regions and DSs were calculated for each cell type (Equation 6) (Fig. 4a,b). We observed that the nuclear deformation distributions of the L929 and hOB nuclei were located in R1-R2, while SH-SY5Y, MCF7 and Saos-2 were in R3-R5 (Fig. 4a). Their DSs were L929: 2.17, hOB: 2.54, Saos-2: 4.02, MCF-7: 3.90 and SH-SY5Y: 3.55 (Fig. 4b). When the deformation scores for each cell type on Control and P4G4 were calculated, only the SH-SY5Y cells had both DS higher than 3; for the others, DS values were lower than 3 on Control. For SH-SY5Y cells the original fluorescence micrographs indicated that these cells grew in clusters preventing their proper assessment with the image pre-processing methods described here (*see* Methods- Image Pre-processing).

On P4G4, hOB and L929 had DS < 3, SH-SY5Y had DS > 3 and MCF7 and Saos-2 had DS > 3.5 (Fig. 4b). In micrograph z-stacks of L929 and hOB, we observed that nuclei were localized on top of the pillars rather than in between (Fig. 3c,d).

To test the algorithm, 4449 nuclei from the 5 different cell types (2944 Saos-2, MCF7 and SH-SH5Y cancer cells, 1673 hOB and L929 non-cancer cells) were analysed individually on P4G4. Out of the 5 regions described on CV-R plot, R1-R2 signified the non-deformable nuclei (CV ≤ 0.2, R ≤ 0.2) and R3-R4-R5 signified the deformable nuclei (CV > 0.2, R > 0.2). Our analysis with the MIND algorithm identified 2821 of the cells as ‘*Deformed*’ (cells with deformable nuclei) and 1673 as ‘*Non-Deformed*’ (cells with undeformable nuclei). ‘*Non-Deformed*’ cells were mostly fibroblast and osteoblast like cells (66%) while ‘*Deformed*’ ones belonged to cancer cell lines (84%). None of the cell populations completely fell into a single region showing that cells do not consist of homogeneous populations even from the same cell line or culture flask, and nuclear deformability is different among the members of the same population (Fig. 4a).

It can, therefore, be concluded that this method is able to reveal the differences in nuclear deformability of cells in a seemingly homogenous population. These results show that MIND is a promising analytical tool to distinguish different cells types and identify cell heterogeneity within the same cell population by quantifying and categorizing changes in the morphology of nuclei in response to micropatterned substrates.

## Discussion

Micron sized surface features are known to cause changes in cell morphology. Cell nuclei are affected by these surface features and deform. Extent of nucleus deformation in a cell could reflect many of its properties. In this study we showed that extent of nuclear deformation of the cells on a micropatterned surface could be quantified. A micropillar array consisting of 9 surfaces with different pillar dimensions and interpillar spacings was used in the quantification of changes in nuclear morphology. It was observed that nuclear morphology was more sensitive to changes in interpillar spacing than pillar dimensions.

Surface selection results showed that 4 μm gap size (smallest tested) has deformed the cell nuclei the most (Fig. 2). Davidson *et al.* earlier reported their observation on nuclear deformation on 7 μm spaced micropatterned surfaces^13^. Badique *et al.* proposed that surfaces with micropatterned decorations of 5–10 μm interpillar spacing deformed cell nuclei more than those with 2–4 μm interpillar spacing^25^. In contrast, in the present study the interpillar spacings smaller than 8 μm showed more prominent nuclear deformation. An earlier study used micropillar decorated surfaces and concluded that pillar aspect ratio is also influential on nuclear deformation^24^. The present study demonstrated that the aspect ratio of the pillars is also important in inducing nuclear deformation; cells on P4 pillars (aspect ratio 2) showed higher nuclear deformations than P8 (aspect ratio 1) and P16 (aspect ratio 0.5).

After finding the surface for the highest level of nuclear deformation quantification (P4G4), this surface was tested with five different cell types. Each cell type had its individual deformation characteristic and cells originating from cancerous tissues deformed more extensively compared to the other cell types tested such as osteoblast like cells or fibroblasts. Different cell types and cells from a single population show a spectrum of deformation behaviour.

Studies on P4G4 surface showed that Saos-2, MCF-7 and SH-SY5Y cells were more inclined towards nuclear deformation than noncancerous hOB and L929 (Fig. 4). One of the studies showed that metastatic lung, breast and pancreatic cancer cells were 70% softer than benign cells^31^ and cancer cells over all have lower densities^65^. Nucleus stiffness also follows a similar trend; loss of Lamin A/C during carcinogenesis leads to a decrease in the stiffness and an increase in the elasticity of the nucleus^66^.

The MIND approach allows quantification of nuclear elasticity of cell populations and individual cells. Rectangularity and circle variance, which are scale, rotation and translation invariant, were found to be optimal and have sufficient features for quantifying deformations of cells on micropatterned surfaces because the cell populations tested have shown to form distinct clusters in the rectangularity-circle variance analysis (Fig. 3). This information is key to discriminating non-deformed cells from deformed cells, and could be useful in future work, especially in fine-tuning algorithm for specific cell types. In addition to being scale, rotation and translation invariant, use of these two simple descriptors, namely circle variance and rectangularity offers the advantage that they create a 2D space for image analysis. This 2D space can be visualized easily and does not introduce an additional dimensionality reduction step for calculations. Furthermore, they have low computational complexity, allowing for inexpensive hardware implementations. Recent studies in the literature reported that osteosarcoma cells with different metastatic potentials (MG-63 and Saos-2 cell lines) showed nuclear deformations on physically patterned polymeric surfaces^13^,^14^. This osteosarcoma cell nucleus deformation was explained as a reflection of the increased flexibility and deformability of cancer cells^28^,^29^. In this study, we quantitatively present that, nuclei of different cell types, including Saos-2 human osteosarcoma cells respond differently to substrate micropatterns.

The gold standard for tissue diagnosis is pathological examination of biopsy, cytology or surgical specimens for their morphological changes. However, even this method carries intrinsic sources of error and the assessment bias. The studies show that cytology is not sensitive but specific (identifies true negative results better), and therefore, biopsy is preferred as a conventional method^67^. On the other hand, using the same data set, and a set of standardized evaluation criteria, agreement between six pathologists was only 58% for breast cancer diagnosis^68^. This demonstrated the need for alternative, device-based (computer-assisted) morphology analysis systems such as^69^,^70^,^71^. Two such examples are the commercially available products FocalPoint (Becton Dickinson)^72^, and the ThinPrep (Hologic)^73^. A study found that these computer assisted cervical screening devices were not cost effective when compared to manual readings in addition to having significantly reduced sensitivity^74^.

In this study, a set of micropatterned surfaces were tested to induce maximum deformation of nucleus and an image recognition algorithm was designed to capture and quantify deformations that were induced by the micropatterns in individual cells and populations easily and rapidly using human and computer vision. Classification or identification of cells from images is often done using hundreds of features (e.g., see refs 71 and 75). The extraction of some of these images can become computationally expensive as the number of cells increase. Extracting fewer features, in this study only two, has multiple advantages. First, throughout all stages of processing, data can be plotted and visualized in two-dimensions, without the use of dimensionality reduction techniques. This eliminates the loss of potentially meaningful data through projection into lower dimensions. Second, the amount of computational burden is easily manageable. Current implementation of the code using MATLAB on a 2.3 GHz Intel Core i7 processor takes under 0.1 second per cell. This makes analysis using inexpensive hardware available on even computers with low processing power possible. Further, because the algorithm is composed of commonly used image processing functions, it can be quickly reprogrammed in any programming language. A recent study showed that Nucleus Form Factor (corresponds to a modified version of the Circle Variance) and Solidity (corresponds to a modified version of the Rectangularity) are the morphological properties that are affected the most by surface topography confirming our choice of morphological parameters^76^.

There is no direct way to compare conventional methods and the system proposed here due to differences in cell types and sources used. Additionally, the relation between nuclear deformability and neoplastic nature of a typical cell is not straightforward. However, a rough comparison of the sensitivity of the methods can be made if these aspects are kept in mind. The method proposed in this study showed 81% sensitivity and 72% specificity at the single cell level (4449 individual nuclei analysed, 2944 were from cancer cell lines and 1550 were from non-cancerous cell sources; 2821 were identified as deformable and 1673 were identified as undeformable) and 100% sensitivity and 85% specificity for population-wise analysis (5 cell populations analysed) (calculated using data from Figs 3 and 4). On the other hand, as was reported above^68^, interobserver reproducibility of pathological diagnosis is still under discussion in many other studies (e.g. refs 77 and 78) and automated morphometric diagnosis is proven to be a promising tool to overcome problems with interobserver reproducibility^79^,^80^.

The MIND approach allows testing of cells from various tissues as long as they are adherent. This feature makes it possible to evaluate a great number of cells in a certain population in a high throughput manner. In its current state, MIND is a promising approach for measuring the range of nuclear deformability in any given cell population allowing comparisons of single cells and populations from multiple sources.

## Additional Information

**How to cite this article**: Ermis, M. *et al.* A high throughput approach for analysis of cell nuclear deformability at single cell level. *Sci. Rep.* **6**, 36917; doi: 10.1038/srep36917 (2016).

**Publisher’s note**: Springer Nature remains neutral with regard to jurisdictional claims in published maps and institutional affiliations.

## Supplementary Material

Supplementary Information
