# Supplementary material for: A high throughput approach for analysis of cell nuclear deformability at single cell level
Source: Sci Rep. 2016 Nov 14;6:36917. doi: 10.1038/srep36917 (PMC5107983; doi:10.1038/srep36917)
Supplement: Supplementary Information [file srep36917-s1.pdf]

SUPPLEMENTARY INFORMATION

A high throughput approach for analysis of cell nuclear deformability at single cell level

Menekse Ermis[1, 2], Derya Akkaynak[3], Pu Chen[4], Utkan Demirci[4, *], and Vasif Hasirci[1, 2, 5,*]

[1]BIOMATEN, METU Centre of Excellence in Biomaterials and Tissue Engineering, 06800, Ankara, Turkey

[2]METU Department of Biomedical Engineering, 06800, Ankara, Turkey

[3]Department of Mechanical Engineering, Massachusetts Institute of Technology, 77 Massachusetts Avenue,

Cambridge, MA 02139, USA

[4]Bio-Acoustic-MEMS in Medicine (BAMM) Laboratory, Canary Centre at Stanford for Early Cancer Detection,

Department of Radiology, School of Medicine, Stanford University, Palo Alto, CA, 94304, USA

[5]METU Department of Biological Sciences, 06800, Ankara, Turkey

*vhasirci@metu.edu.tr, utkan@stanford.edu

SUPPLEMENTARY INFORMATION

Tables

Supplementary Table 1: Examples of the methods to study cell and nuclear stiffness/deformability in the literature.

Method	*Cell type*	*Studied feature/structure*	*No of cells/ measurements*	*Ref.*
AFM indentation	Benign human breast epithelial cells (MCF-10A)<br>Malignant human breast epithelial cells (MCF-7)	Global cell elasticity (Bulk Young's modulus)	*Not given in text*	*28*
PDMS microchannel-microfluidics device	Benign breast epithelial cells (MCF-10A)<br>Non- metastatic tumor breast cells (MCF-7)	Elongation index, transit velocity in channel	50 cells	*29*
AFM indentation	Non-tumorigenic prostate cells (PZHPV-7)<br>Prostatic adenocarcinoma (PC-3)<br>Metastatic prostate carcinoma (Du 145)<br>Metastatic prostate carcinoma (LNCaP)<br>Normal mammary tissue (A184A1)<br>Breast cancer (T47D)<br>Breast adenocarcinoma (MCF7)	Global cell elasticity (Bulk Young's modulus)	232 indentation in 10x10 µm region	*30*
AFM indentation	Patient pleural effusion samples	Stiffness and adhesion	40 metastatic tumor cells<br>48 normal cells	*31*
Magnetic tweezers assay	Human ovarian cancer cell lines (OVCA429, IGROV, SKOV3, HEY, DOV13, OV2008 and Ovca420)<br>Ovarian cancer stable cells lines (Ovca429Neo, Ovca429TbRIII)	Stiffness and compliance	*Not given in text*	*32*
AFM elastography	Primary neonatal rat alveolar type I and II cells	Elastic map of cells	30 cells, 121 indentations per cell	*51*
AFM indentation	Human neuroblastoma cells (SH-SY5Y)	Stiffness	*Cell number not given in text*, 36 indentations per cell	*52*
Hydrodynamic stretching	Patient pleural effusion samples<br>Mouse embryonic stem cells (mESC)<br>Human embryonic stem cells (hESC)	Cell deformability under hydrodynamic focusing	3427 mESC cells<br>2523 hECS cells	*53*
Micropipette aspiration	Human melanoma cell line (WM35)<br>Metastatic melanoma cell line (Lu1205)	Nuclear deformation	66 WM 35 cells<br>45 Lu1250 cells	*54*

SUPPLEMENTARY INFORMATION

Figures

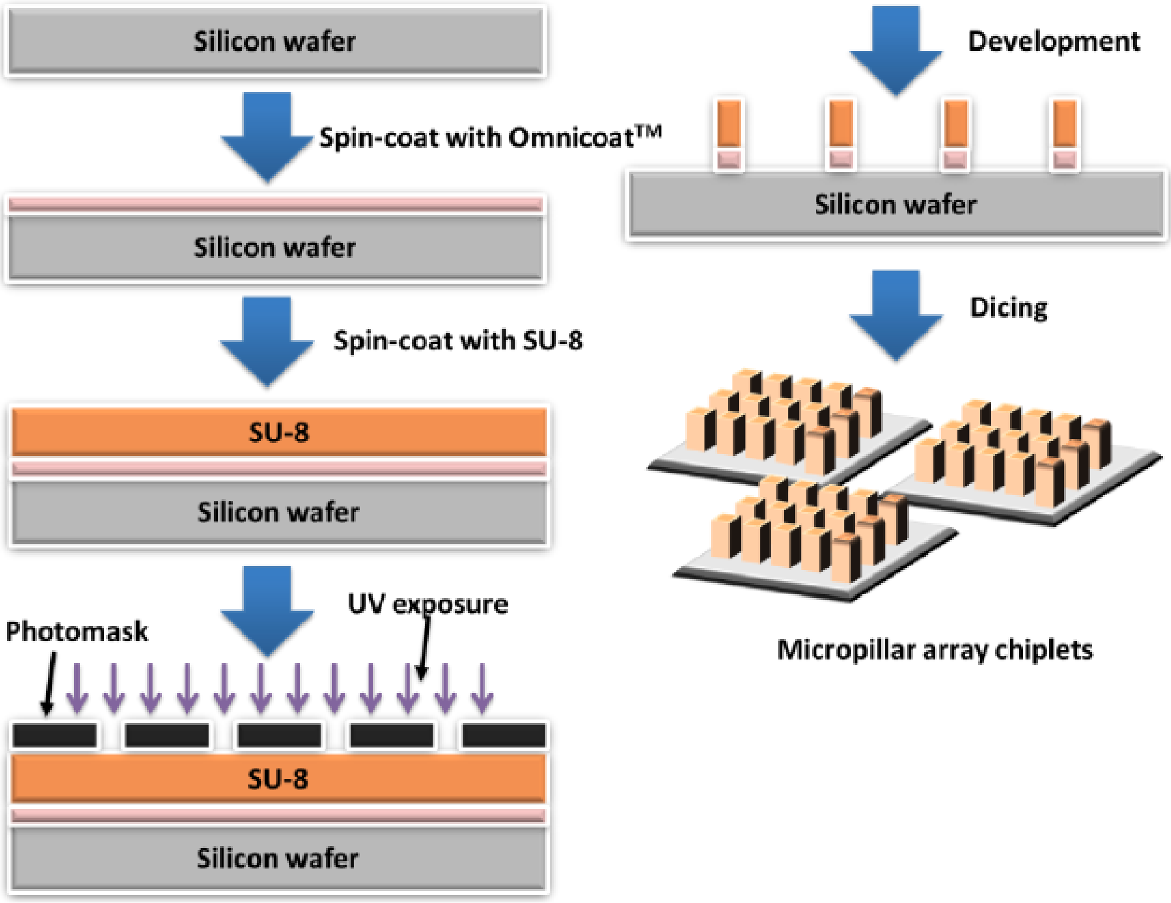

Supplementary Figure 1: Production of SU-8 micropillar array chip.

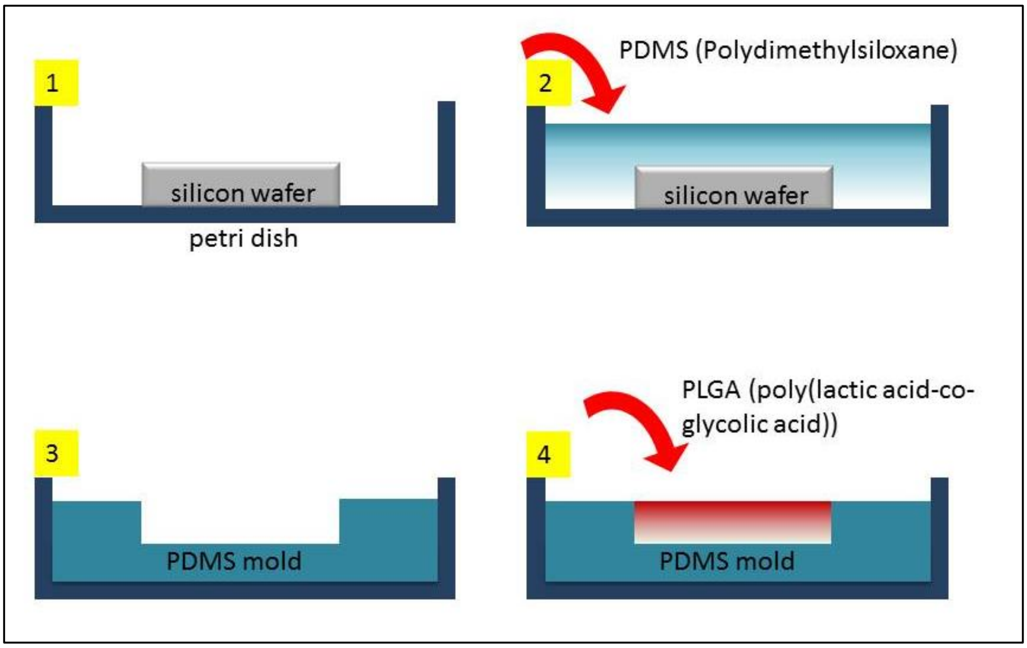

Supplementary Figure 2: Preparation of PDMS mods and poly(lactic acid-co-glycolic acid) (PLGA) (85:15) micropatterned films from SU-8 micropatterned array chiplets.

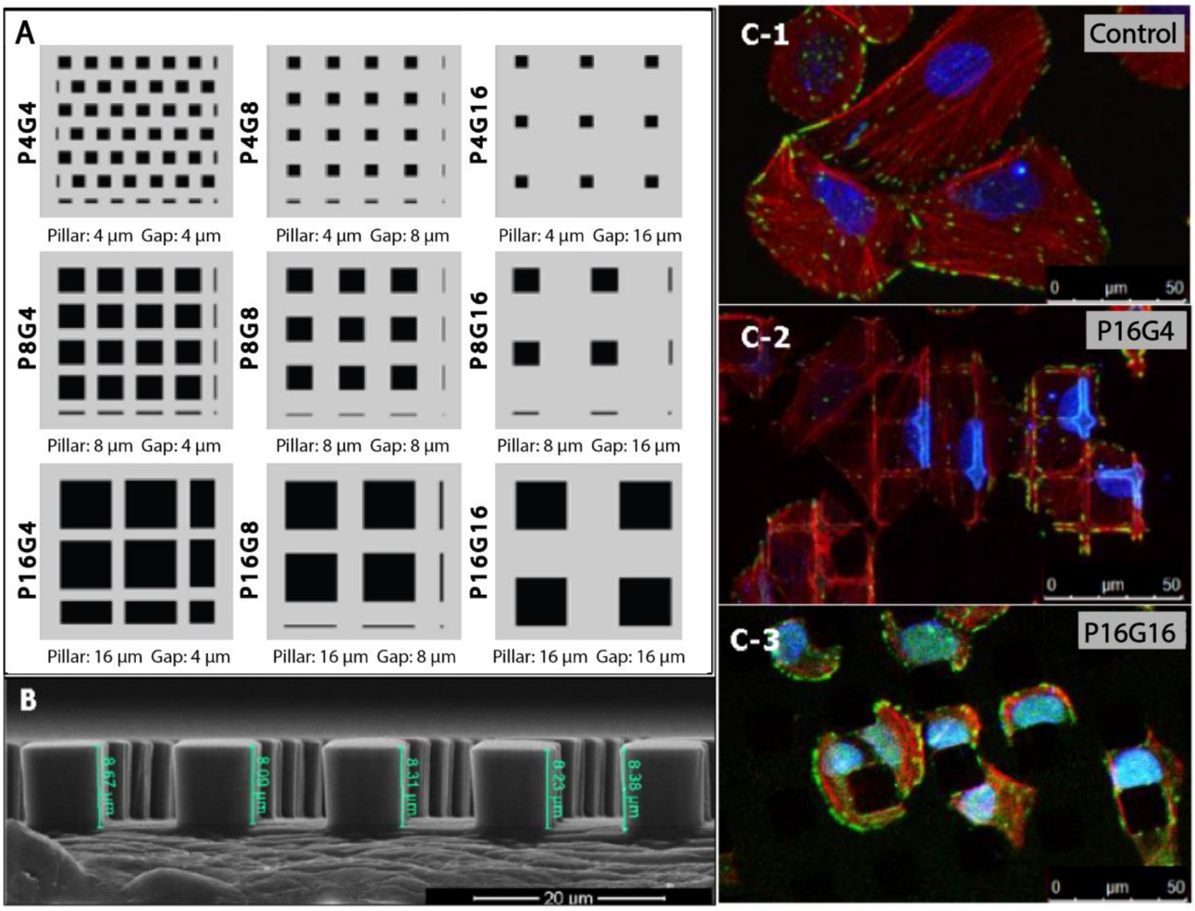

Supplementary Figure 3: Design of the micropillar array chip and the nucleus deformation response of Saos-2 cells to the various pillar and spacings. (a) The array, (b) SEM of a field on the surface (side view), and (c) deformation of Saos-2 nuclei of on some representative surfaces. (a) Top row consist of 4x4 µm pillars, from left to right spacing between the pillars are 4 µm, 8 µm, 16 µm. Middle row consist of 8x8 µm pillars, from left to right spacing between the pillars are 4 µm, 8 µm, 16 µm. Bottom row consists of 16x16 µm pillars, from left to right spacing between the pillars are 4 µm, 8 µm, 16 µm. (b) shows the side view of the pillars. Average pillar heights were determined as 8.3±0.2 µm. (c1) Saos-2 osteosarcoma cells on Control surfaces show round-to-oval nuclei. (c2 and c3) Different levels of nuclear deformation were observed on P16G4 and P16G16, with more extensive deformation on the narrower gap (G4). Stains: Blue: nucleus/anti-LMNA, Red: cytoskeleton/Alexa-Phalloidin, Green: focal adhesions/anti-Paxillin).

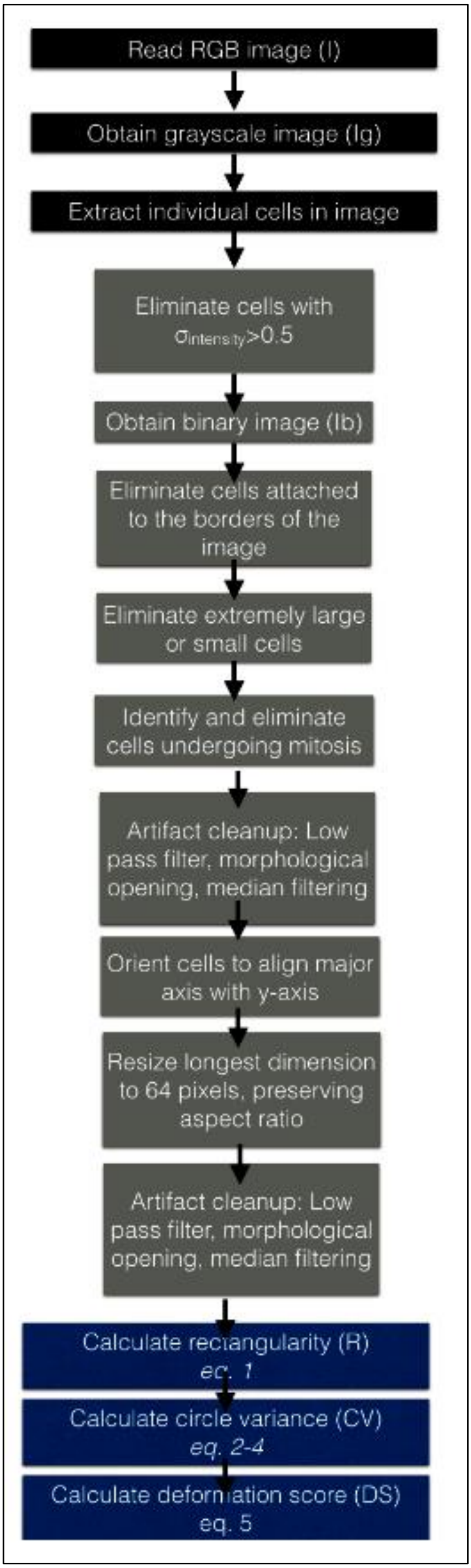

Supplementary Figure 4: High-level flow of image processing algorithm.
